# Evaluating predation of *Megalurothrips usitatus* (Thysanoptera: Thripidae) by *Amblyseius swirskii* (Acari: Phytoseiidae) and *Neoseiulus cucumeris* (Acari: Phytoseiidae)

**DOI:** 10.1093/jee/toag076

**Published:** 2026-03-31

**Authors:** Mariana Monteiro de Souza Barros, Alexandra M Revynthi, Hugh A Smith

**Affiliations:** Institute of Food and Agricultural Sciences, Gulf Coast Research and Education Center, University of Florida, Wimauma, FL, USA; Institute of Food and Agricultural Sciences, Tropical Research and Education Center, University of Florida, Homestead, FL USA; Institute of Food and Agricultural Sciences, Gulf Coast Research and Education Center, University of Florida, Wimauma, FL, USA

**Keywords:** thrips, predation, predatory mites, biological control, integrated pest management

## Abstract

*Megalurothrips usitatus* (Bagnall) (Thysanoptera: Thripidae), a major pest of leguminous crops in the Asian tropics, was first reported in Florida in 2020 and has since spread across Central America and the Caribbean. To control *M. usitatus*, Florida snap bean growers face higher production costs from increased insecticide use. To achieve more effective and sustainable management of *M. usitatus*, integrated approaches, including biological control, are needed. This study evaluated 2 commercial predatory mites, *Amblyseius swirskii* (Athias-Henriot) and *Neoseiulus cucumeris* (Oudemans) (Mesostigmata: Phytoseiidae), as biological control agents against *M. usitatus* through leaf disk bioassays and growth room cage studies. In the leaf disk bioassays, both predators showed high survival while feeding on *M. usitatus*, with daily oviposition rates of 1.07 for *A. swirskii* and 1.48 for *N. cucumeris*, and consumed 3 to 4 first instar larvae per day. Growth room cage studies evaluated whether these predatory mites could suppress *M. usitatus* on *Phaseolus vulgaris* plants. Weekly evaluations over 4 wk showed that *A. swirskii* significantly reduced *M. usitatus* population by approximately 75% by week 4. However, mean thrips densities remained high, with 101.2 ± 31.2 individuals per plant (mean ± SE), and significant plant damage was observed. *Neoseiulus cucumeris* did not significantly reduce thrips populations compared to control plants. This study provides evidence that *A. swirskii* can reduce *M. usitatus* populations on *P. vulgaris* plants under these conditions, but suppression remained limited, indicating that further work is needed to improve effectiveness.

## Introduction

The invasive thrips species *Megalurothrips usitatus* (Bagnall) (Thysanoptera: Thripidae) is native to and broadly distributed across the Asian tropics, where it causes severe damage to leguminous crops (Fabales: Fabaceae) (Chang 1987, Mound and Walker 1987, Tang et al. 2015, Zafirah and Azidah 2018). Its first documented presence in the United States occurred in March 2020, specifically in Florida’s Miami-Dade County, where specimens were collected from *Phaseolus vulgaris* L. (Soto-Adames 2020). Since its introduction in Florida, *M. usitatus* has expanded into parts of Central America and the Caribbean (Gutiérrez 2020, Orozco 2022, Cabrera-Asencio and Jensen 2023, Campos et al. 2023, Racancoj et al. 2023, Rodríguez-Arrieta et al. 2023), representing a serious risk to regional food security due to its impact on beans, a dietary staple in these areas. *Megalurothrips usitatus* primarily feeds and resides in legume flowers but also damages leaves and pods (Chang 1987, Tang et al. 2015), and causes harmful effects on plant growth, including curled leaves, bud drop, stunted development, and malformed pods (Orozco 2022).

Florida’s snap bean (*P. vulgaris*) production spans approximately 15,000 hectares, with a total value of approximately $123 million USD in 2023 (USDA NASS 2024). In this context, Ivey et al. (2024) highlighted the substantial impact of *M. usitatus* on snap bean production in southern Florida, particularly within the Homestead Agricultural Area (HAA) in Miami-Dade County. During the winter months, this region is a crucial source of fresh snap beans for the country. Before the arrival of *M. usitatus*, snap bean growers in South Florida utilized chemical control to manage *Thrips palmi* Karny (Thysanoptera: Thripidae) and *Bemisia tabaci* MEAM1 (Hemiptera: Aleyrodidae) (Ivey et al. 2024). However, *M. usitatus* damages foliage and flowers earlier in the season compared to *T. palmi*, prompting snap bean growers to adjust their management strategies. This adjustment involves applying insecticides earlier and more frequently to mitigate *M. usitatus* infestations.

The predominant strategy for managing this insect pest has been through chemical control applications (Peter and Govindarajulu 1990, Yasmin et al. 2019, Ivey et al. 2024). Although insecticides can provide short-term suppression, their repeated use is associated with environmental concerns, non-target effects, and pest resurgence (Tang et al. 2016). Moreover, species of thrips, including *M. usitatus*, have shown a propensity for developing insecticide resistance (Tang et al. 2016, Fu et al. 2022). Control efficacy is further constrained by thrips’ biological traits, including rapid reproduction and their tendency to reside within flowers, which reduce exposure to foliar applications (Liu et al. 2018). Given these factors, exploring alternatives to insecticidal control for managing *M. usitatus* is crucial.

The primary arthropod predators utilized in pest management of thrips species include *Orius* spp. (Hemiptera: Anthocoridae) and predatory mites, including the Phytoseiidae family (Wu et al. 2018). *Amblyseius swirskii* (Athias-Henriot) (Mesostigamata: Phytoseiidae) and *Neoseiulus cucumeris* (Oudemans) (Mesostigmata: Phytoseiidae) have emerged as significant players in greenhouse pest management, demonstrating efficiency in controlling various small arthropods, including whiteflies, broad mites (*Polyphagotarsonemus latus*), and thrips (Bolckmans et al. 2005, Messelink et al. 2005, Calvo et al. 2011, Onzo et al. 2012, Xiao et al. 2012).

To assess the potential of these phytoseiid predators in managing *M. usitatus* populations, this study integrates 2 experimental approaches. First, a laboratory leaf disk bioassay was conducted to evaluate the effectiveness of *A. swirskii* and *N. cucumeris* in attacking and preying upon *M. usitatus* first instar larvae (L1), and to assess impacts on oviposition and survival when fed a diet consisting of *M. usitatus* L1.

Subsequently, a growth room cage study was conducted using *P. vulgaris* plants infested with *M. usitatus* to simulate more realistic environmental conditions and evaluate predator efficacy at the plant level. While small arenas provide useful initial assessments, trials conducted at the whole-plant level offer the advantage of capturing the complexity of plant architecture, which can influence predator–prey interactions (Schmidt 2014). Plant traits, including trichomes and domatia, may hinder predator mobility or shelter prey (Sabelis and Van Rijn 2005); also, floral structures can provide pollen, an important alternative food resource for generalist predators like *A. swirskii* and *N. cucumeris* (Kreiter et al. 2003). Incorporating these features makes it possible to evaluate more realistically the capacity of predatory mite populations to suppress *M. usitatus*.

This study was guided by the following hypotheses. Under laboratory leaf disk conditions, if *A. swirskii* and *N. cucumeris* are able to utilize *M. usitatus* L1 as a prey resource, then both predators should feed on larvae, and diets consisting of *M. usitatus* L1 should sustain predator survival and egg production. Additionally, under more complex conditions, if these predatory mites are able to suppress *M. usitatus* populations, then predator presence in whole-plant cage studies should reduce thrips infestation on bean plants over time compared to thrips-infested plants lacking predators.

By combining these 2 experimental approaches (laboratory assays and plant-level evaluations), this research increases understanding of the effectiveness of *A. swirskii* and *N. cucumeris* as biological control agents against *M. usitatus*. These findings will contribute to the development of more sustainable integrated pest management strategies, with the goal of reducing chemical reliance and enhancing biological control in leguminous crops. Together, these experiments define the baseline performance of predatory mites against *M. usitatus.*

## Materials and Methods

### *Megalurothrips usitatus* Colony

To initiate the colony, *M. usitatus* individuals were collected from lablab bean (*Lablab purpureus* (L.) Sweet) farms in the Homestead Agricultural Area (HAA), located in Miami-Dade County, Florida (within 80.5563 W, 25.5246 N, and 80.4309 W, 25.4340 N), between January and October 2021. At each collection site, 50 flowers were gathered, placed in Ziploc bags, and then stored in a secured cooler for transportation to the University of Florida’s Gulf Coast Research and Education Center (GCREC) near Tampa, Florida. A subset of collected adults was slide mounted to confirm species identification. To the naked eye, *M. usitatus* is similar in appearance to *Frankliniella insularis* (Franklin) (Thysanoptera: Thripidae), which is common in lablab bean.

Collected flowers were placed in insect cages (BugDorm-2120 F insect rearing tents; W60 × D60 × H60 cm, 160 μm aperture woven mesh; MegaView Science Co., Ltd, Taiwan) with potted bean plants (*P. vulgaris*) of the ‘Caprice’ variety (Harris Seeds, Rochester, New York) in a secured, climate-controlled growth room (25 ± 1.5 °C, 56 ± 7% RH, and L16:D8 photoperiod). Once the field-collected individuals were established on potted bean plants inside the cages, the thrips were aspirated using a handheld aspirator into glass-eyedroppers in groups of 10 and examined under the microscope to confirm species. Confirmed *M. usitatus* individuals were transferred to new cages for the establishment of the colony in a growth room designated solely for the thrips colony.

For colony maintenance, Caprice beans were cultivated in 10 cm pots filled with Jolly Gardener Premium Potting Mix (Oldcastle Lawn & Garden, Inc., Maine, United States) in a separate insect-free growth room, with approximately 7 to 10 seeds planted in each pot. These bean plants were nurtured for a 3-wk period, and watered 3 times a week and fertilized with Osmocote fertilizer (15-9-12 NPK) (The Scotts Company, Marysville, Ohio). Every week, 1 pot with several bean plants was transferred to each *M. usitatus*-infested cage as a food source and oviposition substrate for the colony.

### Phytoseiid Mite Stock Colony

To initiate the colonies of *A. swirskii*, and *N. cucumeris*, predators were ordered from Sierra Biologicals (Lyndonville, New York), a commercial insectary producing and supplying biological control agents. The rearing arenas were maintained at 26 ± 2 °C, 70 ± 5% RH, and L13:D11 photoperiod in a growth chamber. The rearing arenas (Gutierrez 1985) were constructed by placing black plastic plates (80 × 150 mm) on top of a sponge soaked in deionized (DI) water, placed within plastic containers. Cotton wool was used to cover the edges of the plastic plates, ensuring contact with the sponge and water, thereby maintaining a constant moisture level. This setup served a dual purpose as it prevented mite escape and acted as a water source. Sheltered areas were created by cutting and folding strips of the same material as the black plastic plates (approximately 15 × 25 mm), providing hiding places for the mites. For oviposition, a few cotton threads were placed under the house-shaped strips. The mites were fed with decapsulated dry cysts of the brine shrimp *Artemia franciscana* Kellogg (Anostraca: Artemiidae) from BioBee, Kibbutz Sde Eliyahu, Israel, and with cattail (*Typha latifolia* L.) pollen from Biobest, Westelo, Belgium.

### Predatory Mite Egg Cohorts

Both experiments (leaf disk bioassay and cage study) required gravid female predatory mites, aged 3 to 4 d into adulthood. To maintain a consistent supply of predators under these specific conditions, new rearing setups were established weekly using eggs from the main colony. These mites were reared specifically for use in the experiments, under the same growth chamber conditions (26 ± 2 °C, 70 ± 5% RH, 13:11 h L:D photoperiod), but provisioned exclusively with first and second-instar larvae of *M. usitatus* to ensure familiarity with the prey.

## Experiment 1: Leaf Disk Bioassay

For logistical reasons, namely limited space within the growth chamber, the 2 mite species (*A. swirskii* and *N. cucumeris*) were tested separately. Each predator species was evaluated using 22 female replicates. The experiment was conducted on 4 separate dates, which served as blocks (2 dates for *A. swirskii* and 2 for *N. cucumeris*). For each species, 4 treatments were tested. The first treatment involved confining gravid female predatory mites with five *M. usitatus* L1, replaced every 24 h so that 5 fresh larvae were available in each interval. This density was selected to provide sufficient prey while avoiding rapid leaf overexploitation and allowing consistent treatment comparisons. The remaining 3 treatments were controls. The first control group consisted of a single gravid female predatory mite without access to any food source, allowing the observation of its mortality and fertility under such conditions. The second control group consisted of 5 *M. usitatus* L1 without any predators present, allowing the assessment of their survival in the absence of predators. Finally, the third control group involved providing cattail pollen as a food source for gravid female predatory mites, allowing comparison of their survival and fertility when fed on *M. usitatus* L1 versus pollen. The predatory mites used in this experiment were selected as described in section (Predatory Mite Egg Cohorts) Also, to standardize hunger levels, predators were starved for 24 h before the beginning of the experiment. *Megalurothrips usitatus* L1 were obtained from the laboratory colony described in the section (*Megalurothrips usitatus* Colony).

The experimental arenas were constructed using a modified Munger cell design (Overmeer 1985, Argolo et al. 2020). Each arena consisted of 3 acrylic plates measuring 7.5 × 25 mm with a depth of 5.0 mm. Two plates had a centered hole with a diameter of 1.5 cm. The assembly involved placing a solid plate at the bottom, followed by a moistened cotton tissue. On top of the cotton, a bean leaf disk with a 2 cm diameter was positioned with the abaxial surface facing upward. The 2 plates with holes were then stacked on top of the leaf disk, separated by a wire mesh (Grainger 400 wire mesh, 38.5 μm opening). The solid plate and the first plate with a hole enclosing the leaf disk were secured with tape, while all 3 plates were held together using binder clips.

Each Munger cell arena comprised an experimental unit and was prepared by placing 5 *M. usitatus* L1 alongside 1 young gravid female predator (*A. swirskii*, or *N. cucumeris*, depending on the trial) using a fine paintbrush. Using a stereomicroscope, the interactions between the mites and the thrips larvae were observed for the initial 30 min, and the number of prey consumed was recorded, as well as the time until the mite’s first attack, a measure of aggressiveness. At specific time intervals of 24, 48, and 72 h, additional groups of 5 first-instar thrips larvae were introduced into each arena. If the predator did not feed on all 5 thrips from the previous day, the remaining thrips were removed from the arena. The assessments at 24, 48, 72, and 96-h time points included scoring the number of prey consumed, the number of eggs laid by the predator, and the mortality of both predator and prey.

### Statistical Analysis

#### Thrips Mortality

This analysis investigated the effects of 2 fixed factors, treatment and time interval, as well as their interaction, on the response variable, the number of dead thrips larvae (thrips mortality). Treatment consisted of thrips larvae either exposed or not exposed to the predator, and the time intervals were 0.5, 24, 48, 72, and 96 h. “Block” was included as a random effect to account for experiment replicates conducted on different dates.

For each predator species, thrips mortality was modeled as a function of treatment, time interval, and their interaction, using a generalized linear mixed-effects model (GLMM) with a negative binomial error distribution and a log link function, implemented using the lme4 package (v1.1-29) (Bates et al. 2015) in R-version 4. 2. 2. (R Core Team 2022). The negative binomial distribution was selected to account for overdispersion in the count data. The model structure was specified as *DeadThrips ∼ Treatment × Time interval + (1 | Block)*. Fixed effects were evaluated using a Type II analysis of deviance based on Wald χ^2^ tests. Model assumptions and fit were evaluated using residual diagnostics. Post hoc multiple comparisons were conducted using the estimated marginal means with a Tukey adjustment for the probabilities from the *emmeans* package (v1.7.3) (Lenth and Piaskowski 2025).

#### Oviposition

The effect on the fertility of *A. swirskii* when exposed to different treatments (provisioned with thrips larvae, pollen, or deprived of any food source) was analyzed using the number of eggs laid as the response variable. Treatment was included as a fixed effect, and “Block” was included as a random effect.

For *A. swirskii*, oviposition data were fitted to a GLMM with a negative binomial error distribution and a log link function. The negative binomial distribution was used to account for overdispersion in egg count data. The model structure was specified as *MiteEgg ∼ Treatment + (1 | Block)*.

The same procedure was performed to analyze treatment effects with *N. cucumeris*. However, a GLMM with a Poisson error distribution and a log link function was used for this analysis, as egg count variance was comparable to the mean and no overdispersion was detected. The same model structure described above was used.

For both species, fixed effects were evaluated using a Type II analysis of deviance based on Wald χ^2^ tests. Model assumptions and fit were evaluated using residual diagnostics. Post hoc multiple comparisons were conducted using estimated marginal means with Tukey adjustment, using the *emmeans* package.

#### Predator Survival

A time-to-event analysis using the “survreg” function from the “survival” package (Therneau 2024) was conducted to determine the time to mortality of *A. swirskii* across the same 3 treatments: *A. swirskii* provided with thrips larvae, pollen, or deprived of any food source. Time to mortality (hours) was used as the response variable, representing the survival of the predatory mite under each treatment regime. A parametric survival model with logistic error distribution (based on the Akaike Information Criterion [AIC]) was used. Treatment was included as a fixed effect, and “Block” was included as a clustering variable to account for non-independence among experimental replicates. The model structure was specified as *Surv(Time, Censor) ∼ Treatment*. Censoring was applied to predatory mites that survived until the end of the experiment (ie 96 h).

A similar analysis was conducted for *N. cucumeris*, with the only difference being that the AIC identified the lognormal error distribution as the best fit.

## Experiment 2: Cage Study

### Bean Plants

Bean plants (*P. vulgaris* var ‘Caprice’) (Harris Seeds, Rochester, New York) were cultivated in 10 cm pots in a separate insect-free growth room. Although approximately 5 to 7 seeds were initially sown in each pot to ensure germination, only the largest single seedling was retained per pot and grown for the experiments. These bean plants were grown for a 4-wk period and watered 3 times a week and fertilized with Osmocote fertilizer (15-9-12 NPK) (The Scotts Company, Marysville, Ohio).

### Preparing *M. usitatus* for Experimental Use

To obtain adult thrips of the same age (2 to 3 d old) for cage study experiments, a clean Caprice bean plant was placed inside a cage with adult *M. usitatus* from the main colony for 24 h. After this period, the plant was removed and immersed in a bucket of water for about 10 min, or until all remaining adult thrips were removed or dead. This ensured that only eggs laid during the 24 h remained on the plant. The plant was then transferred to a new cage and kept for 12 d, after which newly emerged adults were ready for use in experiments.

### Cage Study

Two experiments were conducted, 1 for *A. swirskii* and 1 for *N. cucumeris*, which were tested separately for logistical reasons. Each experiment was repeated twice in time (blocks). Within each block, 24 cages were used and allocated equally to 2 treatments. The treatments were defined as follows: (i) bean plants infested with *M. usitatus* and not exposed to predators (no-predator, NP) and (ii) bean plants infested with *M. usitatus* with predatory mites introduced 4 d after thrips infestation (predator, P). To reduce the risk of cross contamination, cages assigned to each treatment were positioned on opposite sides of the enclosed growth room, which was maintained at 25 ± 1.5 °C, 56 ± 7% RH, and an L16:D8 photoperiod. Predator introduction was not spatially randomized across cages, because the experiment was conducted in a controlled growth room with uniform light, temperature, and humidity, thereby minimizing the potential for positional environmental gradients. Predatory mites were selected as described in section (Predatory Mite Egg Cohorts).

Four-week-old bean plants, as described in “Bean Plants” section, were individually placed inside insect cages (BugDorm-2120 F insect rearing tents (W60 × D60 × H60 cm, 160 μm aperture woven mesh, MegaView Science Co., Ltd, Taiwan), including a plastic bottom and mesh on the sides. Inside the cage, the plant pot was positioned on a circular platform (100 mm diameter) situated within a container (150 × 300 mm) filled with soapy water, ensuring that the plant remained elevated and avoided direct contact with the soapy water. This setup provided isolation and prevented mites from escaping the plant.

In both treatments, plants were infested with five adult female *M. usitatus*, selected as described in “Preparing *M. usitatus* for Experimental Use” section. They were aspirated into glass eye droppers and released directly inside the experimental cages containing the plants. A 4-day interval was implemented to allow the thrips to establish on the plants, lay eggs, and for the F1 first instar thrips larvae to emerge. Following this waiting period, 5 gravid female predatory mites (*A. swirskii* or *N. cucumeris*) were selected as previously described in “Predatory Mite Egg Cohorts” section. The mites were aspirated into pipette tips, which were then tied to the plants of the Predator (P) treatment only using a twist-tie.

Sampling was initiated 1 wk after the introduction of predatory mites in the Predator (P) treatment and occurred weekly for 4 consecutive weeks (7-, 14-, 21-, and 28-d post predator release). Each week, a destructive sample of 3 whole plants per treatment was conducted. The plants were enclosed in plastic bags and cut at the base near the soil, while ethanol was sprayed to immobilize and capture any flying adult thrips. In the laboratory, using a stereomicroscope, all parts of the plant (leaves, flowers, buds, pods, stem) underwent examination to record numbers of *M. usitatus* adults (males and females), and first and second instar larvae. Floral structures were submerged in ethanol and carefully opened to ensure that concealed thrips were fully recovered and accurately counted. *Megalurothrips usitatus* undergoes 2 feeding larval instars that reside on the vegetative parts of the plant, whereas the non-feeding prepupal and pupal stages develop in the soil. Therefore, pupal stages were not monitored. Moreover, all motile stages of mites (adults, larvae, protonymphs and deutonymphs), as well as any non-target insects present on the plant, were recorded and placed in ethanol.

### Statistical Analysis for Cage Studies

#### Thrips Population

The response variable was the mean of all *M. usitatus* stages (first and second instars, adult females and males) observed in weekly plant samples (ie 6 plants per weekly sample, considering the experiment was repeated twice). Treatment (No-Predator and Predator), time interval (sample week), and their interaction were treated as fixed factors. Blocks (representing replicates of the experiment) were incorporated as a random factor. Data were analyzed using a GLMM with a negative binomial error distribution and a log link function, selected to account for overdispersion in thrips count data. The model structure was specified as *MotileThrips ∼ Treatment × Week + (1 | Block).* Fixed effects were evaluated using a Type II analysis of deviance based on Wald χ^2^ tests. Model assumptions and fit were evaluated using residual diagnostics. When a significant treatment-by-week interaction was detected, post hoc comparisons were conducted within each week using estimated marginal means with Tukey adjustment, implemented using the *emmeans* package.

#### Predator Mite Population

This analysis was conducted separately for *A. swirskii* and *N. cucumeris*. To analyze the effect of time interval (week) on the total number of motile mites (including larvae, protonymphs, deutonymphs, and adult stages), data were analyzed using a GLMM with a negative binomial error distribution and a log link function. Week was included as a fixed effect, and block (representing experimental replicates) was included as a random effect. The negative binomial distribution was selected to account for overdispersion in mite count data. The model structure was specified as *MotileMite ∼ Week + (1 | Block)*. Fixed effects were evaluated using a Type II analysis of deviance based on Wald χ^2^ tests, and model assumptions and fit were evaluated using residual diagnostics.

#### Growth Rate (Thrips Population Dynamics)

To investigate the population dynamics of thrips, we calculated the intrinsic rate of increase (rm) following the exponential growth formulation of Birch (1948), using the equation:


$$rm=\;\frac{\ln\left(Nt/N_{0}\right)}{T},$$

where Nt is the number of thrips at the present week, $N_{0}$ is the number of thrips in the previous week, and T is the number of weeks. This measures the change in population size per week. Under predator treatments, rm was interpreted as a relative index of population change over time rather than as a true intrinsic growth rate, given that predator-induced suppression violates the assumption of exponential population growth. This analysis aimed to estimate the rate at which the population of thrips (including first and second instars, and adult males and females) grew or declined over the observation period.

A linear mixed-effects model (LMM) with a Gaussian distribution was fitted using the *glmmTMB* package (Brooks et al. 2017) to analyze the growth rate (rm) of thrips, with treatments (Thrips with predator present and Thrips without predator), time interval (week), and their interaction as fixed factors, and block (representing experimental replicates) as random factor. The model structure was specified as *rm ∼ Treatment × Week + (1 | Block)*. Fixed effects were evaluated using a Type II analysis of deviance based on Wald chi-square tests. Model assumptions and fit were evaluated using residual diagnostics, and post hoc comparisons were performed using the *emmeans* package.

## Results

### Experiment 1 Results: Leaf Disk Bioassay

#### *Megalurothrips usitatus* Mortality

**Fig. 1.**
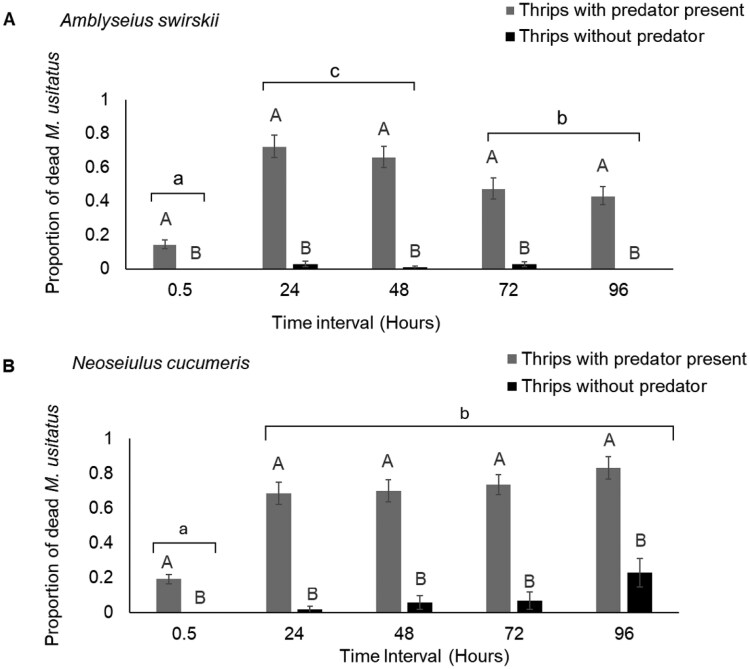
Proportion of dead thrips (mean ± SE) over time (hours) in 2 treatment groups: A) *Amblyseius swirskii* and B) *Neoseiulus cucumeris* provided with 5 *M. usitatus* L1 (grey) and a control group of thrips without predators (black). Significant differences between treatments are indicated by uppercase letters. Different lowercase letters denote statistically significant differences among time intervals (GLMM, *P* < 0.05, *N* = 22).

Both predatory mite species, *A. swirskii*, and *N. cucumeris*, demonstrated an average consumption of approximately one *M. usitatus* L1 within the initial 30 min. Subsequently, they consumed approximately 3 to 4 larvae during each of the successive time intervals of 24, 48, 72, and 96 h. There was a significant increase in thrips larval mortality in the treatment group receiving the predator *A. swirskii* compared to the control, in which the thrips larvae were not exposed to any predator (GLMM: χ^2^ = 104.99, df = 1, *P* < 0.001). Thrips mortality in the control treatment remained near zero across all evaluation periods, with mean proportions ranging from 0 to 0.03, indicating minimal natural mortality in the absence of predators. In contrast, in the predator treatment, the proportion of dead thrips ranged from 0.15 at 0.5 h to 0.73 at 24 h. Additionally, time interval significantly affected *M. usitatus* mortality in the presence of *A. swirskii* (GLMM: χ^2^ = 77.977, df = 4, *P* < 0.001), with mortality being significantly lower at 0.5 h compared to later time points. At 24 and 48 h, mortality increased and remained consistent, before declining at 72 and 96 h (Fig. 1A). The interaction between treatment and time interval did not significantly affect the thrips mortality (GLMM: χ^2^ = 3.58, df = 4, *P* = 0.47).

For *N. cucumeris*, the presence of the predator led to a significantly higher mortality of thrips larvae compared to the control (GLMM: χ^2^ = 203.1, df = 3, *P* < 0.001). Thrips mortality in the control treatment remained low throughout the evaluation period, with mean proportions ranging from 0 to 0.23, whereas in the predator treatment, the proportion of dead thrips increased from 0.19 at 0.5 h to 0.83 at 96 h. Time interval also significantly influenced *M. usitatus* mortality (GLMM: χ^2^ = 110.44, df = 5, *P* < 0.001), with mortality being significantly lower at 0.5 h before increasing and remaining constant for the rest of the evaluation period (Fig. 1B). The interaction between treatment and time interval did not significantly affect thrips mortality (GLMM: χ^2^ = 7.47, df = 4, *P* = 0.11).

#### Oviposition of *A. swirskii* and *N. cucumeris* During Predation on *M. usitatus*

**Fig. 2.**
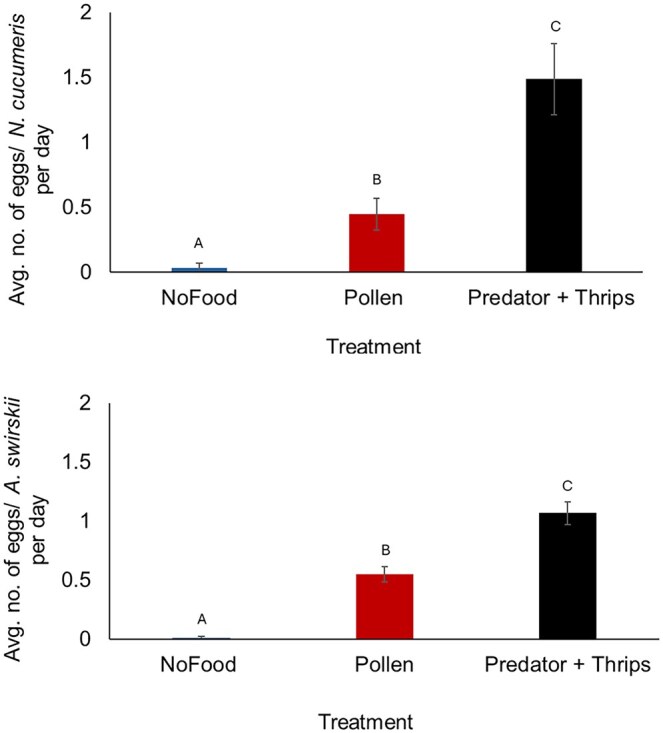
Oviposition rates (mean ± SE) of *A. swirskii* and *N. cucumeris* under different treatment groups, including predator provided with 5 *M. usitatus* larvae (Predator + Thrips), Typha pollen (Pollen), and absence of food (NoFood). Different uppercase letters indicate significant differences among treatments (GLMM, *P* < 0.05, *N* = 22).

Treatment significantly influenced the oviposition of *A. swirskii* (GLMM: χ^2^ = 30.655, df = 2, *P* < 0.001) and of *N. cucumeris* (GLMM: χ^2^ = 75.3, df = 2, P < 0.001). For *A. swirskii*, mean oviposition (mean ± SE) was lowest in the no-food treatment (0.01 ± 0.01 eggs per female per day), intermediate in the pollen treatment (0.55 ± 0.06 eggs), and highest when feeding on *M. usitatus* L1 (1.07 ± 0.10 eggs). Similarly, for *N. cucumeris*, mean oviposition increased from 0.03 ± 0.04 eggs in the no-food treatment to 0.45 ± 0.12 eggs in the pollen treatment and 1.49 ± 0.27 eggs when feeding on *M. usitatus* L1 (Fig. 2).

#### Survival of *A. swirskii* and *N. cucumeris* While Preying on *M. usitatus* L1

**Fig. 3.**
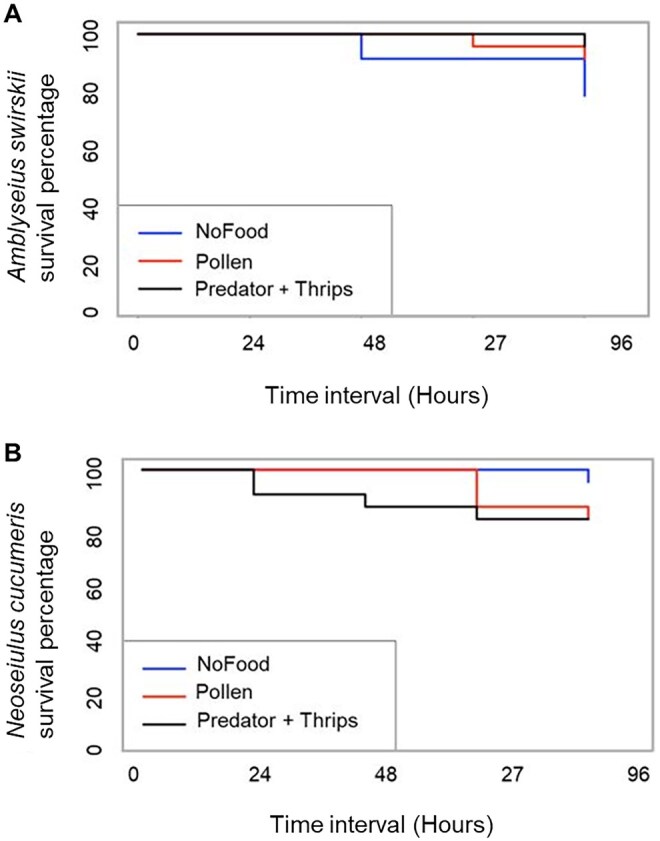
Survival percentage (%) of (A) *A. swirskii* and (B) *N. cucumeris* across time intervals (Hours). Treatment groups included predator provided with 5 *M. usitatus* larvae (Predator + Thrips), Cattail pollen (Pollen), and predator deprived of food (NoFood).

Both *A. swirskii* (Fig. 3A) and *N. cucumeris* (Fig. 3B) maintained consistently high survival rates across all treatments (*M. usitatus* L1, cattail pollen, no food). For *A. swirskii*, survival ranged from 86% to 100% across evaluation periods. Similarly, *N. cucumeris* survival ranged from 86% to 100%. The treatments did not have a significant impact on the survival of *A. swirskii* (χ^2^ = 3.53, df = 2, *P* = 0.17) or *N. cucumeris* (χ^2^ = 3.816, df = 2, *P* = 0.15).

### Experiment 2 Results: Cage Study

#### Impact of Predator Introduction on *M. usitatus* Population Over 4 Wk

**Fig. 4.**
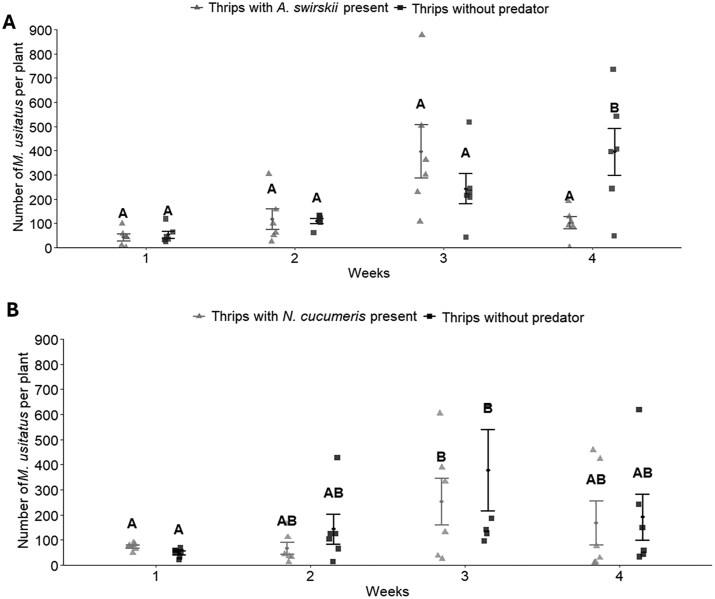
Number of *Megalurothrips usitatus* per plant over 4 wk when (A) *A. swirskii* and (B) *N. cucumeris* were released. Two treatments were evaluated: Thrips with predator present (grey) and Thrips without predator (black). Points represent individual plant-level observations. Bars indicate mean ± SE. Predators were introduced 4 d after the thrips infestation, and destructive samples were taken weekly. For (A), different letters indicate significant differences among treatment × week combinations resulting from a significant interaction between treatment and week (GLMM, *P* = 0.02, *N* = 24). For (B), letters indicate significant differences among weeks based on a significant main effect of week only (GLMM, *P* = 0.001, *N* = 24).

The introduction of the predator *A. swirskii* significantly impacted *M. usitatus* populations in week 4, as indicated by a significant interaction between treatment (Thrips without predator (Control) and Thrips with *A. swirskii* present) and time interval (week) GLMM: χ^2^ = 9.99, df = 3, *P* = 0.02). During weeks 1, 2, and 3, thrips, population did not differ significantly between treatments. However, in week 4, the control treatment had significantly higher thrips population compared to the treatment receiving *A. swirskii* (post hoc Tukey comparisons, all *P* > 0.05) (Fig. 4A).

When *N. cucumeris* was introduced as the predator, no significant treatment effect on *M. usitatus* populations was observed (GLMM: χ^2^ = 1.63, df = 1, *P* = 0.2). Similarly, there was no significant interaction between treatment and time interval (GLMM: χ^2^ = 5.13, df = 3, *P* = 0.16). However, a significant effect of time interval alone was detected (GLMM: χ^2^ = 15.47, df = 3, *P* = 0.001). Thrips population in week 1 was significantly lower than in week 3 (post hoc Tukey comparison, *P* < 0.05) (Fig. 4B).

### Predator Population Over the Weeks

**Fig. 5.**
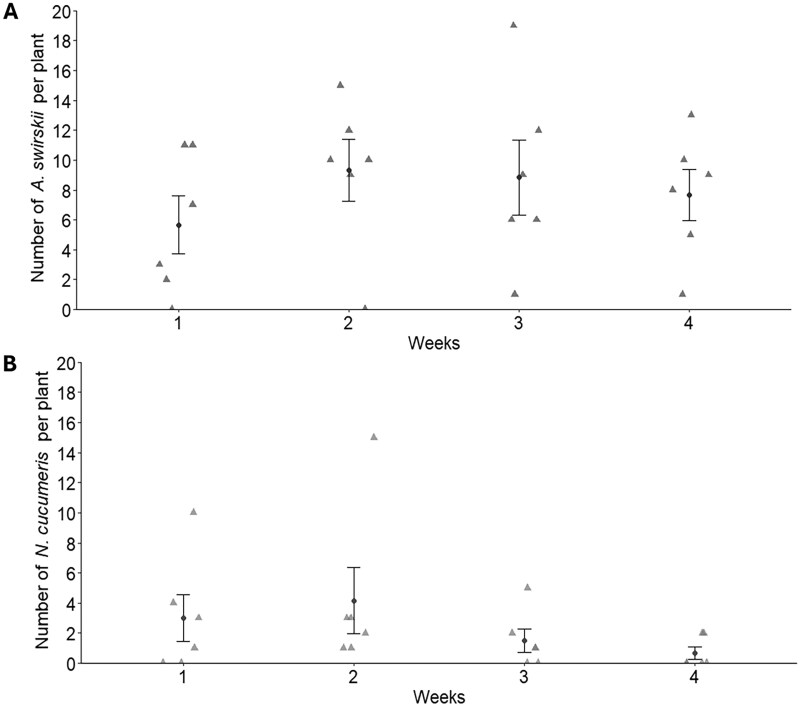
Number of motile predatory mites per plant over 4 wk in cage experiments when (A) *Amblyseius swirskii* and (B) *Neoseiulus cucumeris* were released. Points represent individual plant-level observations. Bars indicate mean ± SE. Predators were introduced 4 d after the thrips infestation, and destructive samples were taken weekly.

The total number of motile *A. swirskii* did not significantly vary across the four weeks of evaluation (GLMM: χ^2^ = 1.67, df = 3, *P* = 0.64) (Fig. 5A). In week 1, the mean number of motile mites per plant was 5.6 ± 1.9 (mean ±SE). This number slightly increased to 9.3 ± 2.06 mites in week 2. In weeks 3 and 4, the mean number of predators was 8.83 ± 2.52 and 7.6 ± 1.7 mites, respectively.

Similarly, the number of motile *N. cucumeris* did not show significant variation over the four weeks of evaluation (GLMM: χ^2^ = 3.1, df = 3, *P* = 0.38) (Fig. 5B). During week 1, the average number of motile mites was 1.8 ± 1.3 (mean ± SE). This number increased slightly to 2.08 ± 1.7 mites in week 2. By week 3, the mean number decreased to 0.75 ± 0.6 mites, and further declined to 0.3 ± 0.3 mites in week 4, indicating that *N. cucumeris* failed to establish under the cage conditions.

### *Megalurothrips usitatus* Growth Rate Across Weeks With and Without Predators

**Fig. 6.**
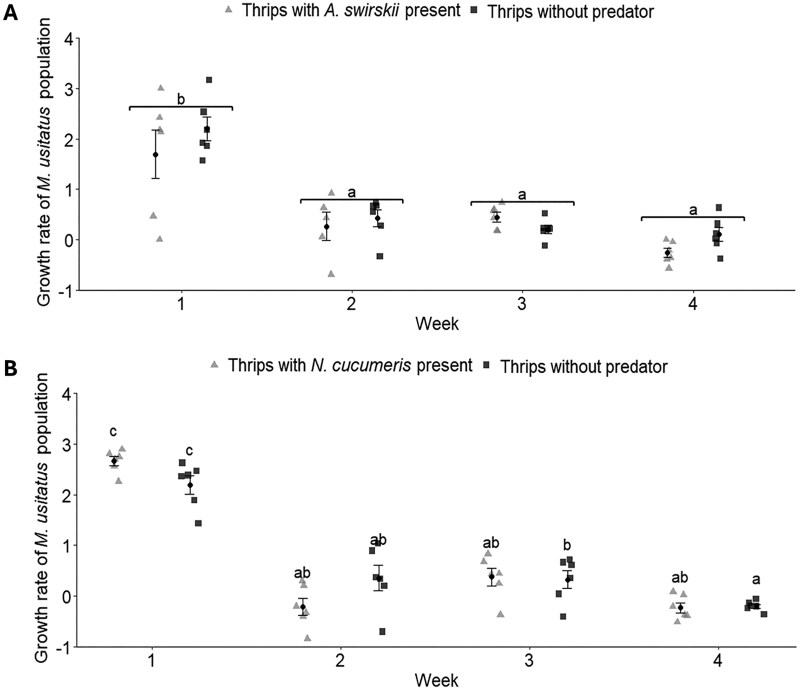
Population growth rate (*r*m) of *Megalurothrips usitatus* over 4 wk when (A) *Amblyseius swirskii* and (B) *Neoseiulus cucumeris* were released. Two treatments were evaluated: Thrips with *A. swirskii* present or Thrips with *N. cucumeris* present (grey triangles) and Thrips without predator (black squares). Points represent individual plant-level observations. Bars indicate mean ± SE. Mites were released on day 4 with samples taken weekly. For (A), different lowercase letters indicate significant differences among weeks (GLMM, *P* < 0.05, *N* = 24). For (B), letters indicate significant differences from the interaction between treatment and week (GLMM, *P* < 0.05, *N* = 24).

*Megalurothrips usitatus* growth rate (rm) parameter was not significantly affected by the introduction of *A. swirskii* to the Predator (P) treatment (GLMM: χ^2^ = 1.75, df = 1, *P* = 0.19). Similarly, the interaction between the time interval (week) and treatment was not significant (GLMM: χ^2^ = 5.07, df = 4, *P* = 0.28). However, the time interval (week) alone had a significant effect on growth rate (GLMM: χ^2^ = 157.27, df = 4, *P* < 0.001). Specifically, week 1, where the growth rate is based on the initial number of *M. usitatus* released on the plants (5 individuals), exhibited a significantly higher mean growth rate of 1.95 ± 0.13 (mean ± SE) compared to the rest of the weeks. The mean growth rates for weeks 2, 3, and 4 were 0.35 ± 0.14, 0.33 ± 0.13, and –0.08 ± 0.13, respectively, which are not significantly different from each other (Fig. 6A).

The growth rate of *M. usitatus* was significantly influenced by the addition of *N. cucumeris* in the Predator (P) treatment, with this effect varying across weeks, with a significant interaction between treatment and time interval (GLMM: χ^2^ = 16.4, df = 4, *P* = 0.002). In week 1, both treatments exhibited markedly elevated growth rates, with the P treatment reaching 2.66 ± 0.14 (mean ± SE) compared to 2.19 ± 0.14 in the NP treatment; these values were significantly higher than in all subsequent weeks. From week 2 onward, growth declined and remained low or negative (week 2: (P) −0.21 ± 0.14; (NP) 0.36 ± 0.14; week 3: (P) 0.38 ± 0.14; (NP) 0.33 ± 0.14; week 4: (P) −0.23 ± 0.14; (NP) −0.2 ± 0.14). Pairwise comparisons within each week did not detect significant differences between treatments, indicating that the significant interaction reflects differences in temporal trajectories rather than consistent within-week treatment contrasts (Fig. 6B).

## Discussion

*Megalurothrips usitatus* poses a significant threat to leguminous crop production, yet its biological control, particularly the use of phytoseiid mites for pest suppression, has received little attention. This underscores the significance of the findings discussed here, as they provide insights into the potential and limitations of commercially available phytoseiid predators as biocontrol agents of *M. usitatus*.

Both predators exhibited similar predation rates in laboratory bioassays, consuming comparable numbers of *M. usitatus* larvae daily. *Amblyseius swirskii* consumed an average of 3.58 first-instar larvae daily over 96 h, while *N. cucumeris* consumed 3.73 larvae daily. These rates are notably higher than those reported in previous studies for other thrips species, such as second-instar *Scirtothrips dorsalis* (Arthurs et al. 2009) or larvae (not specified if L1 or L2) of *Thrips palmi* and *Frankliniella schultzei* (Kakkar et al. 2016), where daily consumption ranged from 1.5 to 2.75 larvae. The higher consumption rates observed in this study suggest that *M. usitatus* L1 are suitable as prey for both predators in laboratory conditions, but prey suitability alone did not translate into strong population-level suppression in the cage studies.

In the cage study, the introduction of *A. swirskii* on *P. vulgaris* plants 4 d after infestation with *M. usitatus* resulted in a significant decrease in the thrips population only by the fourth week, after 28 d, with an approximate 75% reduction compared to the control. However, the remaining thrips population was still high, with 101.2 ± 31.2 individuals per plant (mean ± SE) (Fig. 4A). In contrast, *N. cucumeris* did not significantly reduce thrips populations over the 4-week evaluation period. These results demonstrate that high prey consumption observed in laboratory bioassays did not translate directly into effective population suppression under plant-based conditions, indicating that factors beyond prey suitability constrained predator performance.

In the cage study, predator performance was evaluated under plant-based conditions where multiple biological and environmental constraints may have shaped predator–prey interactions. These include the timing of predator release relative to prey establishment, potential limitations in alternative food resources such as pollen, structural traits of the host plant, and possible prey defensive behaviors. These constraints were not directly quantified and therefore represent limitations of the study, which should be considered as possible contributing factors when interpreting the delayed or limited suppression observed.

Specific plant structures, including trichomes and leaf domatia, can influence predator movement and provide refuge for prey, shaping predator–prey interactions in plant-based systems (Sabelis and Van Rijn 1997). Despite the small size of phytoseiid mites, their capacity to access thrips hiding sites within complex plant structures remains poorly understood (Sabelis and Van Rijn 1997). Experimental studies have shown that increased trichome density can reduce walking speed, delay predation, and decrease predation frequency in *N. cucumeris* (Madadi et al. 2007). For *A. swirskii*, trichome density does not appear to affect the time required to locate prey but can reduce the frequency of prey encounters (Buitenhuis et al. 2014). In addition, some plant structures may indirectly benefit generalist predators by retaining pollen, which can serve as an alternative food source (Kreiter et al. 2003). Together, these findings highlight how host plant morphology can shape predator–prey interactions (Cortesero et al. 2000) and provide important context for interpreting predator performance under cage conditions.

The influence of environmental complexity on predator–prey interactions underscores the importance of evaluating multiple factors when assessing the potential of biocontrol agents. Beyond prey consumption, population-level suppression depends on a predator’s ability to survive and maintain populations over time. Biological traits, such as reproductive performance and survival rates, play a critical role in determining a predator’s ability to establish and sustain populations in varying conditions.

Both *A. swirskii* and *N. cucumeris* exhibited consistently high survival rates across all treatments throughout the leaf disk bioassay. Notably, survival remained steady even in the absence of food, which suggests that both predators endured a 96-h plus starvation period of 24 h, totaling a 5-day period without a food source. This endurance highlights an important adaptation for resource scarcity, suggesting ecological resilience that could enhance biological control efficacy in environments with fluctuating prey availability.

Fecundity directly influences the predator’s establishment on the crop, affecting its ability to increase in population to keep up with the prey’s population growth. Tang et al. (2015) recorded peak daily production of first instar larvae greater than 12 with sexual reproduction, and greater than 16 with parthenogenesis on snap bean. In the leaf disk bioassays, when evaluating fecundity, daily oviposition rates for *A. swirskii* and *N. cucumeris* were 1.07 ± 0.1 and 1.48 ± 0.2 eggs (mean ± SE), respectively, when provided with *M. usitatus* as a food source. In contrast, Delisle et al. (2015) recorded higher fecundity rates when *N. cucumeris* was fed *F. occidentalis*, observing an average of 2.6 eggs daily, while *A. swirskii* averaged around 1.9 eggs under comparable conditions. Furthermore, the findings of a study conducted by Wimmer et al. (2008) reported lower daily oviposition rates, averaging 0.92 eggs per *A. swirskii* fed on *F. occidentalis* and 0.99 eggs per *A. swirskii* fed on *T. tabaci* at similar environmental conditions. These contrasting oviposition rates across different studies suggest that predatory mites respond differently in reproductive performance when exposed to different food sources. Their daily oviposition may vary significantly depending on multiple factors, including differences in prey/food and environmental conditions.

In the present study, the relative humidity in the growth rooms averaged 56 ± 7%, which may have been suboptimal for predatory mite establishment. These fluctuating oviposition rates might be one of the factors reflecting the limited population growth of both predators in the cage studies. Although reproduction was sustained, as evidenced by the presence of immature stages of the predators, their populations remained low throughout the experiment. Other factors likely contributed to this outcome, such as the lack of an alternative food source.

These mites utilize alternative food sources like pollen to supplement their diet (Van Rijn and Tanigoshi 1999). Besides feeding on small arthropods, both *A. swirskii* and *N. cucumeris* can feed and develop on multiple pollen sources (Van Rijn and Tanigoshi 1999, Goleva and Zebitz 2013). In this study, pollen availability in the cage system was not quantified, and limited availability due to early peak bloom may have affected phytoseiid juveniles, which can rely on pollen as an alternative food source during development, particularly when their smaller size makes preying on thrips larvae challenging (Sabelis and Van Rijn 1997). Under these conditions, variation in pollen availability may have been one of several factors shaping predator population dynamics in the cage study, as both predator species remained at low densities.

Implementing artificial pollen supplementation in the crop could enhance predator establishment and increase their densities. A potential concern is that several thrips, including *M. usitatus*, could benefit from the added pollen. However, studies have shown that this is not necessarily the case. In fact, improvements in thrips biological control have been observed with alternative food supplementation, leading to increased pest suppression over time (Deere et al. 2024). Additionally, the authors found no evidence of adverse effects of pollen on thrips control. Further supporting the role of pollen in predator establishment, Kutuk and Yigit (2011) observed that applying artificial pollen to sweet pepper plants before the flowering period strengthened *A. swirskii* populations, enhancing control of *F. occidentalis* infestations.

As generalist Type III predators (McMurtry et al. 2013), *A. swirskii* and *N. cucumeris* can prey on multiple pest species (Calvo et al. 2015). Given the varying prey availability in snap bean fields, other significant pests are also relevant. According to Ivey et al. (2024), in addition to *M. usitatus*, snap bean growers encounter *T. palmi* and *B. tabaci* infestations. Calvo et al. (2011) found that *A. swirskii* effectively suppressed *B. tabaci* and *F. occidentalis* individually, but when both pests were present, mite populations increased significantly. Similarly, Messelink et al. (2008) observed a 15-fold increase in *A. swirskii* densities when both *F. occidentalis* and the greenhouse whitefly (*Trialeurodes vaporariorum* Westwood (Hemiptera: Aleyrodidae)) were present, attributing this increase to the benefits of a mixed diet on predators’ juvenile survival and development. These findings suggest that *A. swirskii* and *N. cucumeris* could thrive in snap bean fields where multiple pests coexist, potentially enhancing their biological control efficacy by leveraging the availability of alternative prey.

In the cage study, predator introduction was timed to ensure prey availability at release. Predators were introduced 4 d after *M. usitatus* infestation, corresponding to the period required for oviposition and emergence of first instar larvae, which were the focal prey stage evaluated in the laboratory assays. This timing was selected because phytoseiid mites are highly mobile and prey availability strongly influences their retention on plants, with dispersal increasing under prey-limited conditions (Lopez 2023). Ensuring the presence of L1 at the time of predator introduction was, therefore, intended to promote predator residence on the plant and to allow evaluation of predator performance once the pest population was already established. Consequently, the cage study represents a suppression rather than a preventative biological control scenario, and predator performance should be interpreted within that context. In contrast, preventative release strategies, particularly when combined with access to alternative food resources, have been shown to support predator establishment and population growth (Deere et al. 2024). Accordingly, growth rate (rm) values estimated for thrips populations in the presence of predators should be interpreted cautiously as comparative indicators of population performance rather than estimates of intrinsic growth potential.

Additionally, a predator’s success in pest control is partially influenced by the prey’s ability to defend itself (Beretta et al. 2024). These authors reported that *Thrips parvispinus* (Karny) (Thysanoptera: Thripidae) and *F. occidentalis* killed *A. swirskii* eggs, while *Echinothrips americanus* Morgan (Thysanoptera: Thripidae) used abdominal swings to resist attacks. The authors also observed that *F. occidentalis* and *T. parvispinus* deterred predators by producing anal droplets, which is a chemical defense. Although antipredator behavior was not assessed in the present study, these findings highlight that prey defense strategies can influence predator–prey interactions in thrips systems. Whether *M. usitatus* exhibits similar defensive behaviors remains unknown and represents an important area for future research when evaluating the effectiveness of phytoseiid predators.

Overall, these findings indicate that although both *A. swirskii* and *N. cucumeris* can effectively consume *M. usitatus* larvae under laboratory conditions, predator establishment and sustained population growth under plant-based conditions remain limiting factors. Future research should focus on identifying strategies that enhance predator retention and reproduction within legume systems to improve suppression outcomes.

## References

[toag076-B1] Argolo PS , Revynthi AM, Canon MA, et al 2020. Potential of predatory mites for biological control of *Brevipalpus yothersi* (Acari: Tenuipalpidae). Biol. Control 149:104330. 10.1016/j.biocontrol.2020.104330

[toag076-B2] Arthurs S , McKenzie CL, Chen J, et al 2009. Evaluation of *Neoseiulus cucumeris* and *Amblyseius swirskii* (Acari: Phytoseiidae) as biological control agents of chilli thrips, *Scirtothrips dorsalis* (Thysanoptera: Thripidae), on pepper. Biol. Control 49:91–96. 10.1016/j.biocontrol.2009.01.002

[toag076-B3] Bates D , Mächler M, Bolker B, et al 2015. Fitting linear mixed-effects models using lme4. J. Stat. Soft. 67:1–48. 10.18637/jss.v067.i01

[toag076-B4] Beretta GM , Zandbergen L, Deere JA, et al 2024. Predator-prey interactions: how thrips avoid predation. Biol. Control 188:105437. 10.1016/j.biocontrol.2023.105437

[toag076-B5] Birch LC. 1948. The intrinsic rate of natural increase of an insect population. J. Anim. Ecol. 17:15–26. 10.2307/1605

[toag076-B6] Bolckmans K , Houten YV, Hoogerbrugge H. 2005. Biological control of whiteflies and western flower thrips in greenhouse sweet peppers with the phytoseiid predatory mite *Amblyseius swirskii* Athias-Henriot (Acari: Phytoseiidae). In: Hoddle MS, editor. Second International Symposium on Biological Control of Arthropods. Vol. 2. p. 555–565.

[toag076-B7] Brooks ME , Kristensen K, van Benthem KJ, et al 2017. glmmTMB balances speed and flexibility among packages for zero-inflated generalized linear mixed modeling. R J. 9:378. 10.32614/RJ-2017-066

[toag076-B8] Buitenhuis R , Shipp L, Scott-Dupree C, et al 2014. Host plant effects on the behaviour and performance of *Amblyseius swirskii* (Acari: Phytoseiidae). Exp. Appl. Acarol. 62:171–180. 10.1007/s10493-013-9735-1 24037505

[toag076-B9] Cabrera-Asencio I , Jensen CE d 2023. First report of the exotic species *Megalurothrips usitatus* (Thysanoptera: Thripidae), pest of Fabaceae, in Puerto Rico. Fla Entomol. 106:267–269. 10.1653/024.106.0410

[toag076-B10] Calvo FJ , Bolckmans K, Belda JE. 2011. Control of *Bemisia tabaci* and *Frankliniella occidentalis* in cucumber by *Amblyseius swirskii*. BioControl 56:185–192. 10.1007/s10526-010-9319-5

[toag076-B11] Calvo FJ , Knapp M, van Houten YM, et al 2015. *Amblyseius swirskii*: what made this predatory mite such a successful biocontrol agent? Exp. Appl. Acarol. 65:419–433. 10.1007/s10493-014-9873-0 25524511

[toag076-B12] Campos OJC , Monroy AC, Arrieta JAR, et al 2023. New report of the exotic species *Megalurothrips usitatus* (Thysanoptera: Thripidae) infesting three commercial legumes in Nayarit, Mexico. Fla Entomol. 105:316–318. 10.1653/024.105.0409

[toag076-B13] Chang NT. 1987. Seasonal abundance and developmental biology of thrips *Megalurothrips usitatus* on soybean in the southern area of Taiwan. Plant Prot. Bull. 29:165–173.

[toag076-B14] Cortesero AM , Stapel JO, Lewis WJ. 2000. Understanding and manipulating plant attributes to enhance biological control. Biol. Control 17:35–49. 10.1006/bcon.1999.0777

[toag076-B15] Deere JA , Beretta GM, van Rijn PCJ, et al 2024. Does alternative food for predatory arthropods improve biological pest control? A meta-analysis. Biol. Control 198:105605. 10.1016/j.biocontrol.2024.105605

[toag076-B16] Delisle JF , Brodeur J, Shipp L. 2015. Evaluation of various types of supplemental food for two species of predatory mites, *Amblyseius swirskii* and *Neoseiulus cucumeris* (Acari: Phytoseiidae). Exp. Appl. Acarol. 65:483–494. 10.1007/s10493-014-9862-3 25430552

[toag076-B17] Fu B , Tao M, Xue H, et al 2022. Spinetoram resistance drives interspecific competition between *Megalurothrips usitatus* and *Frankliniella intonsa*. Pest Manag. Sci. 78:2129–2140. 10.1002/ps.6839 35170208

[toag076-B18] Goleva I , Zebitz CPW. 2013. Suitability of different pollen as alternative food for the predatory mite *Amblyseius swirskii* (Acari: Phytoseiidae). Exp. Appl. Acarol. 61:259–283. 10.1007/s10493-013-9700-z 23670826

[toag076-B19] Gutierrez J. 1985. Systematics. In: Helle W, Sabelis MW, editors. Spider mites: their biology, natural enemies, and control. Vol. 1A. Elsevier Science Pub. Co. p. 75–90.

[toag076-B20] Gutiérrez YU. 2020. First report for Cienfuegos of *Megalurothrips usitatus* Bagnall (Thysanoptera: Thripidae) in the cultivation of beans (*Phaseolus vulgaris* L.). 5:93–94.

[toag076-B21] Ivey C , De Marchi BR, Beuzelin J, et al 2024. Susceptibility to insecticides of *Megalurothrips usitatus* (Bagnall) and *Frankliniella insularis* (Franklin) (Thysanoptera: Thripidae) infesting *Lablab purpureus* in Florida. Crop Prot. 175:106448. 10.1016/j.cropro.2023.106448

[toag076-B22] Kakkar G , Kumar V, Seal DR, et al 2016. Predation by *Neoseiulus cucumeris* and *Amblyseius swirskii* on *Thrips palmi* and *Frankliniella schultzei* on cucumber. Biol. Control 92:85–91. 10.1016/j.biocontrol.2015.10.004

[toag076-B23] Kreiter S , Tixier M-S, Bourgeois T. 2003. Do generalist phytoseiid mites (Gamasida: Phytoseiidae) have interactions with their host plants? Int. J. Trop. Insect Sci. 23:35–50. 10.1017/S1742758400012236

[toag076-B24] Kutuk H , Yigit A. 2011. Pre establishment of *Amblyseius swirskii* (Athias Henriot) (Acari: Phytoseiidae) using *Pinus brutia* (Ten.) (Pinales: Pinaceae) pollen for thrips (Thysanoptera: Thripidae) control in greenhouse peppers. Int. J. Acarol. 37:95–101. 10.1080/01647954.2010.540081

[toag076-B25] Lenth RV , Piaskowski J. 2025. emmeans: estimated marginal means, aka least-squares means. 10.32614/CRAN.package.emmeans

[toag076-B26] Liu P , Jia W, Zheng X, et al 2018. Predation functional response and life table parameters of *Orius sauteri* (Hemiptera: Anthocoridae) feeding on *Megalurothrips usitatus* (Thysanoptera: Thripidae) Predation functional response and life table parameters of *Orius sauteri* (Hemiptera: Anthocoridae) feeding on *Megalurothrips usitatus* (Thysanoptera: Thripidae). Fla Entomol. 101:254–259. 10.1653/024.101.0216

[toag076-B27] Lopez L. 2023. Meet *Amblyseius swirskii* (Acari: Phytoseiidae): a commonly used predatory mite in vegetable crops. Weber D, editor. J. Integr. Pest Manag. 14:20. 10.1093/jipm/pmad018

[toag076-B28] Madadi H , Enkegaard A, Brodsgaard H, et al 2007. Host plant effects on the functional response of *Neoseiulus cucumeris* to onion thrips larvae. J. Appl. Entomol. 131:728–733. 10.1111/j.1439-0418.2007.01206.x

[toag076-B29] McMurtry JA , Moraes GJD, Sourassou NF. 2013. Revision of the lifestyles of phytoseiid mites (Acari: Phytoseiidae) and implications for biological control strategies. Syst. Appl. Acarol. 18:297–320. 10.11158/saa.18.4.1

[toag076-B30] Messelink GJ , van Maanen R, van Steenpaal SEF, et al 2008. Biological control of thrips and whiteflies by a shared predator: two pests are better than one. Biol. Control 44:372–379. 10.1016/j.biocontrol.2007.10.017

[toag076-B31] Messelink GJ , van Steenpaal S, van Wensveen W. 2005. *Typhlodromips swirskii* (Athias-Henriot) (Acari: Phytoseiidae), a new predator for thrips control in greenhouse cucumber. IOBC/WPRS Bull. 28:183–186.

[toag076-B32] Mound LA , Walker AK. 1987. Thysanoptera as tropical tramps: new records from New Zealand and the Pacific. NZ Entomol. 9:70–85. 10.1080/00779962.1987.9722497

[toag076-B33] Onzo A , Houedokoho AF, Hanna R. 2012. Potential of the predatory mite, *Amblyseius swirskii*, to suppress the broad mite, *Polyphagotarsonemus latus*, on the gboma eggplant, *Solanum macrocarpon*. J. Insect Sci. 12:7. 10.1673/031.012.0701 22962997 PMC3465922

[toag076-B34] Orozco J. 2022. *Megalurothrips usitatus* Bagnall (Thysanoptera: Thripidae), first record of an important new pest in Honduras. Insecta Mundi. 0923:1–4.

[toag076-B35] Overmeer WPJ. 1985. Alternative prey and other food resources. In: Helle W, Sabelis MW, editors. Spider mites: their biology, natural enemies, and control. Vol. 1A. Elsevier Science Pub. Co. p. 131–137.

[toag076-B36] Peter C , Govindarajulu V. 1990. Management of blossom thrips, *Megalurothrips usitatus*, on pigeonpea. Int. J. Pest Manag. 36:312–313. 10.1080/09670879009371495

[toag076-B37] Racancoj MA , Miranda IAA, Aguilar IE, et al 2023. Megalurothrips usitatus, una plaga emergente en el cultivo de frijol en Guatemala. ICTA.

[toag076-B38] Rodríguez-Arrieta JA , Chaves-Barrantes NF, Hernández-Fonseca JC, et al 2023. First report of common bean flower thrips *Megalurothrips usitatus* Bagnall in Costa Rica. Trop. Subtrop. Agroecosyst. 26:7. 10.56369/tsaes.4521

[toag076-B39] Sabelis MW , Van Rijn PCJ. 1997. Predation by insects and mites. In: Lewis T, editor. Thrips as crop pests. CAB International. p. 259–354.

[toag076-B40] Sabelis MW , Van Rijn PCJ. 2005. When does alternative food promote biological pest control? In: Hoddle MS, editor. Proceedings of the Second International Symposium on Biological Control of Arthropods. Vol. 2. p. 428–437.

[toag076-B41] Schmidt RA. 2014. Leaf structures affect predatory mites (Acari: Phytoseiidae) and biological control: a review. Exp. Appl. Acarol. 62:1–17. 10.1007/s10493-013-9730-6 23990040

[toag076-B42] R Core Team. 2022. A language and environment for statistical computing. R Foundation for Statistical Computing.

[toag076-B43] Soto-Adames FN. 2020. Megalurothrips usitatus. Florida Department of Agriculture and Consumer Services Division of Plant Industry. Report No.: FDACS-P-02137.

[toag076-B44] Tang L , Yan K, Fu B, et al 2015. The life table parameters of *Megalurothrips usitatus* (Thysanoptera: Thripidae) on four leguminous crops. Fla Entomol. 98:620–625. 10.1653/024.098.0235

[toag076-B45] Tang L , Zhao H, Fu B, et al 2016. Colored sticky traps to selectively survey thrips in cowpea ecosystem. Neotrop. Entomol. 45:96–101. 10.1007/s13744-015-0334-1 26429578

[toag076-B46] Tang L , Zhao H, Fu B, et al 2016. Monitoring the insecticide resistance of the field populations of *Megalurothrips usitatus* in the Hainan area. Environ. Entomol. 38:1032–1037.

[toag076-B47] Therneau T. 2024. A package for survival analysis in R, R package version 3.7. https://cran.r-project.org/web/packages/survival/vignettes/survival.pdf.

[toag076-B48] [USDA NASS] USD of ANASS. 2024. Vegetables 2023 summary. https://esmis.nal.usda.gov/sites/default/release-files/02870v86p/qz20vd735/ht24z584t/vegean24.pdf

[toag076-B49] Van Rijn PCJ , Tanigoshi LK. 1999. Pollen as food for the predatory mites *Iphiseius degenerans* and *Neoseiulus cucumeris* (Acari: Phytoseiidae): dietary range and life history. Exp. Appl. Acarol. 23:785–802. 10.1023/A:1006227704122

[toag076-B50] Wimmer D , Hoffmann D, Schausberger P. 2008. Prey suitability of western flower thrips, *Frankliniella occidentalis*, and onion thrips, *Thrips tabaci*, for the predatory mite *Amblyseius swirskii*. Biocontrol. Sci. Technol. 18:533–542. 10.1080/09583150802029784

[toag076-B51] Wu S , Tang L, Zhang X, et al 2018. A decade of a thrips invasion in China: lessons learned. Ecotoxicology 27:1032–1038. 10.1007/s10646-017-1864-6 29027089

[toag076-B52] Xiao Y , Avery P, Chen J, et al 2012. Ornamental pepper as banker plants for establishment of *Amblyseius swirskii* (Acari: Phytoseiidae) for biological control of multiple pests in greenhouse vegetable production. Biol. Control 63:279–286. 10.1016/j.biocontrol.2012.09.007

[toag076-B53] Yasmin S , Latif MA, Ali M, et al 2019. Management of thrips infesting mung bean using pesticides. SAARC J. Agric. 17:43–52. 10.3329/sja.v17i2.45293

[toag076-B54] Zafirah Z , Azidah A. 2018. Diversity and population of thrips species on legumes with special reference to *Megalurothrips usitatus*. Sains Malays 47:433–439. 10.17576/jsm-2018-4703-02

